# Epigenetic Liver Damage: Study Reveals Clues Implicating 1,3-Butadiene

**DOI:** 10.1289/ehp.119-a218b

**Published:** 2011-05

**Authors:** Bob Weinhold

**Affiliations:** **Bob Weinhold**, MA, has covered environmental health issues for numerous outlets since 1996. He is a member of the Society of Environmental Journalists

The petroleum-derived substance 1,3-butadiene is a known human carcinogen that is a significant contributor to cancer risk in the United States. There is evidence it causes liver, heart, lung, and hematopoietic cancers in rodents through genetic damage. But some researchers suspect it also may induce changes through other pathways, including epigenetic alterations, which occur when the function of a gene is altered while its DNA sequence remains stable. A new study provides further evidence 1,3-butadiene may indeed cause epigenetic damage **[*EHP*** **119(5):635–640; Koturbash et al.]**.

The authors exposed male C57BL/6J mice to inhaled 1,3-butadiene at two doses, 6.25 ppm and 625 ppm, for 2 weeks (6 hours per day, 5 days per week). The low dose is about 10–100 times higher than typical occupational and ambient exposures, respectively, while the inhalation pathway is considered the most common for human exposure. In the 5 mice exposed at each dose, the researchers found numerous dose-dependent alterations in genes linked with liver function. Compared with controls, mice in the low-dose group had 1 gene with a more than 2-fold increase in expression and 5 with a more than 2-fold decrease in expression. Mice in the high-dose group had 4 genes with a more than 2-fold increase in expression and 13 with a more than 2-fold decrease in expression. The high-dose mice also had a small but significant decrease in body weight.

The authors also found evidence of epigenetic changes that were consistent with altered gene expression in the liver. Changes in the attachment of methyl groups to DNA are a useful marker of epigenetic changes, and the researchers observed significant decreases in 5 markers of methylation in the high-dose group and smaller decreases in 4 of the 5 markers in the low-dose group. High-dose mice also had a roughly 50% decrease in another methylation indicator.

Changes in histones, proteins that help regulate gene expression, also occurred in the high-dose mice, with significant decreases in methylation based on 3 biomarkers. Expression of proteins involved in DNA methylation and histone methylation decreased while expression of a protein involved in histone demethylation increased, consistent with the observed decreases in DNA and histone methylation.

These findings fit with earlier research into epigenetic effects of other substances and are consistent with other mechanistic evidence of how such damage can play a role in the onset of various adverse health effects, the authors say. If additional studies—including studies of female mice, other mouse strains, and other animals—repeat these findings, this would indicate multiple modes of carcinogenicity for 1,3-butadiene and could lead to establishment of specific biomarkers for epigenetic damage that could be used in future toxicity and exposure assessments.

